# The Impact of Podiatric Intervention on the Quality of Life and Pain in Children and Adolescents with Hypermobility

**DOI:** 10.3390/ijerph20176623

**Published:** 2023-08-22

**Authors:** Muhammad Maarj, Verity Pacey, Louise Tofts, Matthew Clapham, Andrea Coda

**Affiliations:** 1 School of Health Sciences, College of Health, Medicine and Wellbeing, The University of Newcastle, Ourimbah 2258, Australia; 2 Narrabeen Sports Medicine Centre, Sydney Academy of Sport, Narrabeen 2101, Australia; 3 Department of Health Sciences, Macquarie University, Macquarie Park 2109, Australia; 4 Hunter Medical Research Institute, New Lambton Heights 2035, Australia; 5 Equity in Health and Wellbeing Research Program, Hunter Medical Research Institute, New Lambton Heights 2035, Australia

**Keywords:** children, fatigue, generalized joint hypermobility, lower limb pain, orthotics, quality of life

## Abstract

The purpose of this study was to evaluate the effect of custom-made orthotics on pain, health-related quality of life (HRQoL), function and fatigue in children and adolescents with generalised joint hypermobility (GJH) and lower limb pain. Fifty-three children aged 5–18 years were fitted with custom-made polypropylene orthotics. Visual analogue scale (VAS) assessed lower limb pain severity, Paediatric Quality of Life Inventory assessed HRQoL and fatigue and six-minute walk test (6 MWT) measured functional endurance at baseline, at 1 month and 3 months post-intervention. A mixed model including a random intercept for participant and a fixed effect for time was used to assess differences in outcomes over time. Fifty-two children completed the study (mean age 10.6-years). Children reported significantly reduced pain (mean VAS reduction −27/100, 95%CI: −33, −21), improved HRQoL (mean total improvement 11/100, 95%CI: 7, −15), functional capacity (mean 6MWT improvement 27 m, 95%CI: 18, −36) and fatigue (mean total improvement 13/100, 95%CI: 9, −17) after 1 month of wearing the custom-made orthotics. From 1 month to 3 months there was further statistically but not clinically significant reduction in pain while benefit on other outcomes was maintained. In this study, children with GJH reported reduced lower limb pain, improved HRQoL, functional endurance and fatigue after a month post-fitting of custom-made orthotics which was maintained over a 3 month period. Orthotics were well-tolerated with no serious adverse events reported.

## 1. Introduction

Generalised joint hypermobility (GJH) defined as Beighton 6/9 or more [1] is the presence of excessive range of movements in multiple joints [2]. This condition is prevalent in approximately 34% of children and adolescents globally [3] and is associated with symptoms in about one in five of these children [4,5]. GJH is mostly an inherited or congenital condition but can also be acquired related to a wide range of inflammatory joint or musculoskeletal disorders [6] and certain neurological disorders such as poliomyelitis [7]. There is also a broad range of inherited connective tissue disorders such as Ehlers-Danlos syndrome and Marfan’s Syndrome that are associated with joint hypermobility with considerable clinical overlap [8]. Generalised Hypermobility Spectrum Disorder is the combination of Beighton 5/9 or greater for post-pubertal and 6/9 or greater for pre-pubertal children and adolescents together with musculoskeletal manifestations including joint pain, pes planus, hindfoot valgus, poor proprioception, joint laxity and instability [9]. 

Children with symptomatic GJH can experience joint laxity or joint instability and reduced strength and endurance during walking and other physical activities [10]. Children also frequently report soft tissue injuries and pain following exercise particularly in the lower limbs [11,12]. Longitudinal studies have shown that children with GJH are more likely than their non-hypermobile peers to experience persistent chronic pain 3 to 5 years later [13,14]. In addition, children with symptomatic GJH can experience functional impairments such as difficulties with motor development [15] and impaired proprioception [16]. Overall symptomatic GJH may negatively impact a child’s participation, including school attendance, poor academic performance [17], reduced quality of life [18,19] and psychosocial function [20].

Exercise programs have been reported to be effective at lowering pain intensity and improving muscle strength and quality of life in children with lower limb pain and GJH [21,22,23]. In addition, a recent systematic review found that in both children and adults with symptomatic hypermobility, conservative management strategies (such as physiotherapy and therapeutic exercise) were superior in reducing pain when compared to no treatment [24]. 

There is emerging evidence to support using prefabricated or off-the-shelf orthotics for improving short-term gait patterns and dynamic balance in children with GJH and lower limb pain [25]. Evidence is limited to two studies in children incorporating pre-fabricated insoles: an unblinded randomised trial and a small cohort study [25,26]. In a randomised controlled clinical trial of 52 children with symptomatic GJH, modified prefabricated insoles worn for 12 weeks improved pain, physical health and function, stair ascent time and transfer and basic mobility functions [26]. However, the Beighton cut-off score to indicate GJH was 4/9 in this study, which is lower than currently established cut-offs for children [1,27]. In addition, this trial included children from 3 to 10 years only, missing the ages of 11–18 years where children’s participation in physical activity and sport increases substantially [26]. Secondly, in a cohort study of 21 children with symptomatic GJH (median age 10 years), prefabricated orthotics were found to immediately improve gait patterns [25]. However, no assessment of pain and fatigue was included [25]. Notably in the adult population, a recent observational cohort study involving 41 adults (mean age 40 years) diagnosed with Ehlers Danlos Syndromes demonstrated significant improvements in foot pain, functionality, fatigue and mental health-related quality of life after 3-months of wearing custom-made orthotics [28]. 

To date, no study has investigated the impact of custom-made orthotics on pain, functioning, fatigue and quality of life in children with GJH and lower limb pain. This study therefore investigated the safety of, and outcomes following, custom-made orthotic use with podiatric recommended footwear in children with GJH and lower limb pain through a before and after intervention only study assessing pain, functioning, fatigue and quality of life over a three-month period.

## 2. Materials and Methods

### 2.1. Research Participants

Ethics was approved by the Executive Committee of the Sydney Children’s Hospitals Network Human Research Ethics Committee (Reference number LNR/18/SCHN/480) and Newcastle University (Reference number H-2019-0221). The trial was registered by the Australian New Zealand Clinical Trials Registry (ACTRN12623000688684). Study participants were recruited from Narrabeen Sports Medicine Centre (Sydney Academy of Sport and Recreation, NSW), the Children’s Hospital at Westmead and local allied health practitioners. 

Children diagnosed with GJH were included if they were aged between 5 and 18 years with a Beighton Score of ≥5/9 (post-pubertal adolescents) and ≥6/9 (children and pre-pubertal adolescents) [1,27], they experienced lower limb pain for at least one month in one or more joints and reported ≥2/10 pain level on the Visual Analogue Scale (VAS) during the week prior to the recruitment. Children with other chronic musculoskeletal, neurological, developmental, behavioural or syndromic conditions unrelated to their GJH were excluded. Also, children with recent physical trauma not related to symptomatic hypermobility, children who wore foot orthotics during the last 6 months prior to this study, children unable to walk or follow instructions, as well as those with contraindications to wearing orthotics (e.g., previous triple arthrodesis surgery), were deemed ineligible for this study.

### 2.2. Procedure

Potential participants were invited to attend the Narrabeen Sports Medicine Centre located in New South Wales, Sydney. On this visit, written consent was obtained from parents or caregivers of children who met the inclusion criteria for this study. Demographic and clinical data including age, gender, height, weight, Beighton score, level of pain and any previously medically diagnosed conditions were then collected. Race and ethnicity were not collected. At the same visit, all outcome measures were recorded followed by a biomechanical assessment and a 3D scan of the participant’s feet by an experienced podiatrist. The fitting appointment was then two weeks after the initial visit to ensure orthotics fit inside the participant’s shoes. Assessments 1 month and 3 months post-orthotic fitting repeated and recorded all outcome measures. 

The Beighton Score [29] was used to determine generalized joint hypermobility, whereby a total of nine points is awarded with one point for each of the following tests: hyperextension of the elbows and knees > +10 degrees on each side, touching tip of the thumb to the forearm on each side, extension of the little finger > 90 at the metacarpophalangeal joint on each side, and touching the floor with both hands while knees are kept straight. The same clinician (MM) performed the Beighton Score for each participant. 

An off-weight bearing scanning CAD/CAM technology with high resolution laser capability of 200 dpi (dots per inch) was used (Konica Minolta Vivid 9i non-contact 3D). This device scans both feet in a supine position in approximately two minutes, enabling the clinician to maintain the child’s subtalar joint (STJ) in a neutral position during the scanning process. The 3D foot scanned images enabled the clinician (MM) to then capture a detailed negative 3D mould of the foot. The de-identified foot scanned data was then shared electronically with an external independent manufacturing laboratory (Virtual Orthotics, Mount Kuring-gai, NSW, Australia). 

To ensure reproducibility of the prescription, a standard approach based on validated biomechanical assessments [30] was utilized which followed clinical protocol recommendations developed based on expert consensus [31]. The custom-made orthotics consisted of a prescription polypropylene shell which was individualized for each patient by an experienced podiatrist. Common prescription features included 10% arch fill, deep heel cup and extrinsic and intrinsic rear foot correction. The biomechanical assessment included foot posture index (FPI), bilateral ankle range of motion and knee position and tibial angles, together with a subjective history to exclude other foot or ankle pathologies. At baseline, the child’s footwear was assessed by MM and biomechanical measurements were recorded by the same clinician (MM). Participants were then provided with recommendations for the most appropriate type of shoes to fit the custom-made orthotics. The footwear types recommended had features of a stable shank and strong heel counter. 

At the fitting appointment, each participant was provided with contact details of the clinic and standard instructions (verbally and in writing) on how to gradually increase wear time in the orthotics from an hour the first day, to two hours the second day, to four hours the fourth day and then all day. Participants were provided with a form to record the weekly duration of orthotic wear indicating the number of hours worn per week in categories of 0–10 h and then in 5 h/week increments till 40+ h. Follow-up was conducted independently by a research assistant not involved in the treatment at 1 month and 3 months post-intervention to re-record the outcome measures. 

### 2.3. Adverse Events

Participants were asked to report any serious adverse events (e.g., injury) and minor side effects such as blistering, skin irritation, footwear and fitting problems between visits via phone to the clinic and at each clinic appointment. The chief investigator (MM) documented details and provided immediate support and advice to the participant in the form of appropriate treatment to address each adverse event. A serious adverse event was defined as any severe pain or disability or physical musculoskeletal injury likely resulting from wearing custom-made orthotics.

### 2.4. Primary Outcome Measures

The primary outcome of pain was measured using the VAS in which self-reported pain was indicated on a 100 mm line ranging from 0 = “no pain” to 10 = “worst possible pain” [32]. The electronic version of VAS (eVAS) which has been recently validated for pain evaluation in children and adolescents with symptomatic GJH was used [33].

HRQoL was assessed with the validated instrument Paediatric Quality of life Inventory (PedsQL) Generic Core Scale which has previously been used for HRQoL assessment in both healthy paediatric populations as well as children with specific conditions [34]. The scale is composed of 23 items and measures four functioning domains: physical, emotional, social and school functioning. A clinically important difference is considered to be a 5-point difference between time intervals [34,35]. The PedsQL is available for children to self-report in different age ranges (5–7; 8–12; and 13–18) and scored out of a possible 100 points with a higher score indicative of better HRQoL. The parent proxy-report of the PedsQL Generic Core scale is also validated and was used to measure changes in the participant’s HRQoL reported by parents [35].

### 2.5. Secondary Outcome Measures

Secondary outcomes included the six-minute walk test (6MWT), PedsQL Multidimensional Fatigue Scale (MFS) and the Patient Global Impression of Change (PGIC). To measure functional endurance capacity based on guidelines from the American Thoracic Society [36,37], the 6MWT which is validated [38] and self-paced over 6 min [36] was performed by each participant. The PedsQL MFS is an 18-item validated measure of fatigue in child self-reported and parent proxy-reported versions consisting of sleep, cognitive function and general fatigue domains [34]. The scale instructions and scoring method/100 is the same as the PedsQL generic core scale with higher scores indicating fewer difficulties [39]. Although not validated in children, this scale has been previously used to measure fatigue in children with symptomatic GJH [11,40]. To measure the perception of the participants of the impact of the custom orthotic intervention since the last visit and assess overall health status, the 7-point PGIC scale ranging from 1 = very much improved to 7 = very much worse was used [41]. Participants were asked to describe any changes in the overall physical and emotional status as well as quality of life related to lower limb pain.

### 2.6. Statistical Analysis 

Sample size calculations used pain as the primary outcome measure with a minimum clinically important difference of 8% in the paediatric population [42]. Therefore, a successful outcome for a participant is defined as a decrease in pain of at least 8 mm from baseline over a one month period. A study of 50 or more patients has at least 80% power at 5% one-sided alpha to detect an increase above this rate.

All statistical analysis were performed using R version 4.2.0 (22 April 2022 ucrt: R Foundation for Statistical Computing, Vienna, Austria). Descriptive statistics including mean with standard deviation (SD) if normally distributed or median with range (min and max) if not normally distributed were used to describe demographics at baseline and for each outcome at baseline, 1 month and 3 months post-intervention. The changes over time were assessed with a mixed model including a random intercept for participant and a fixed effect for time. Estimates of differences from baseline to 1 and 3 months and from 1 to 3 months with 95% confidence intervals (CIs) and *p*-values were calculated. The number and proportion of adverse events and PGIC improvement categories are presented with binomial exact (Clopper-Pearson) 95% CIs. Wilcoxon signed rank test was used to test the difference in wear time between weeks. The wear time categories were re-categorized to make equal 10 h intervals for analysis.

## 3. Results

**Figure 1:**
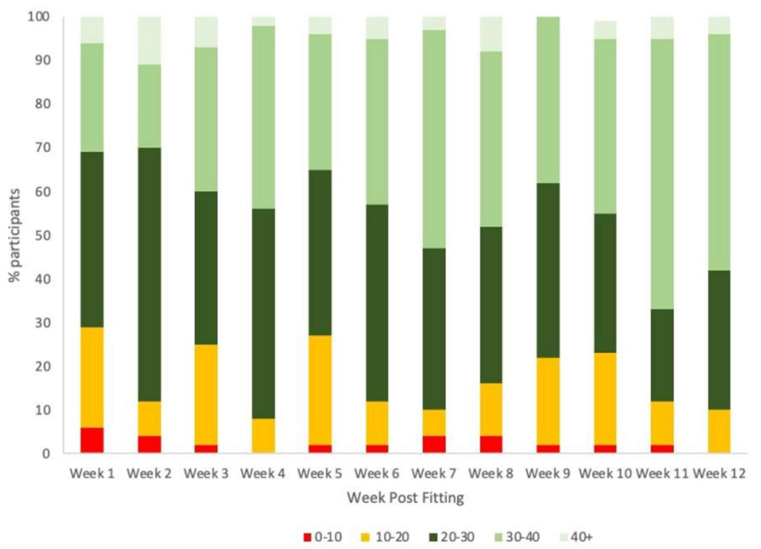
Orthotics wear duration (in hours) post-fitting over the 12–week study period (N = 52).

Fifty-three children with GJH participated in this study with one participant lost prior to the 1 month follow-up due to personal reasons. Most participants were recruited from the Narrabeen Sports Medicine Centre (*n* = 43), with the remainder referred from the Children’s Hospital at Westmead and local allied health practitioners. The demographics and baseline characteristics of participants are summarized in Table 1. Weekly self-reported wear time of the orthotics indicates that participants gradually increased wear time over the trial period, with the majority of participants wearing the orthotics > 20 h per week from week 1, and >30 h/week by week 11 (Figure 1). Significant differences in wear time were demonstrated between week 1 and week 12 (*p* < 0.001) as well as week 2 and week 11 (*p* = 0.027). 

**Figure 2:**
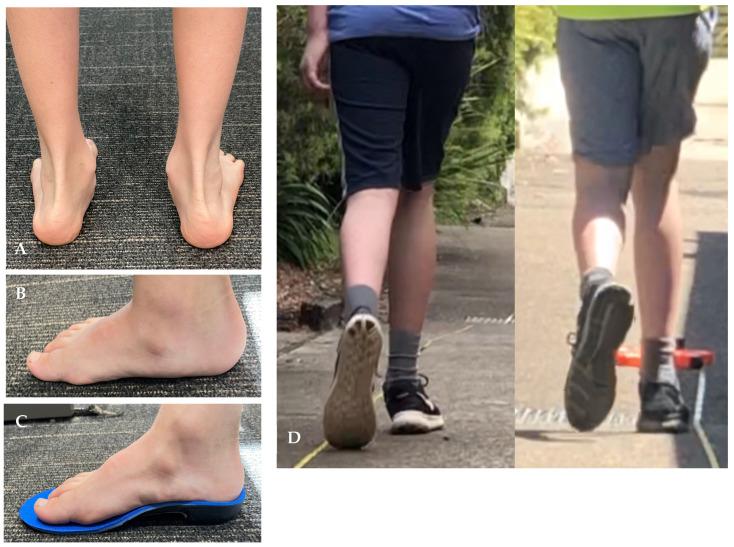
Participant with hypermobility during intervention. (**A**). Typical hypermobile feet. (**B**). Side view of participant foot before wearing the orthotic. (**C**). Side view of participant foot while wearing the orthotic (**D**). Before after podiatric intervention.

No serious adverse effects were reported during the intervention. Seven participants reported one or more minor adverse events during the study. Discomfort was reported by six children at the first fitting appointment. Discomfort in arches of feet (*n* = 3) was addressed with continual education (increase wear time gradually). Blisters during orthotics wear (*n* = 2) was resolved by adjusting the polypropylene shell area and applying a self-adhesive (ENGO) blister patch to the orthotic blister area to reduce shear forces. Immediate heel pain in one participant was resolved by reducing the rearfoot correction. At 1 month one of the participants that was instructed to repeat the gradual wear in the orthotics process still developed an arch blister and this was addressed by applying an ENGO blister patch. All reported adverse events had resolved by the completion of study at the 3 month timepoint with no discomfort reported by any participants. Figure 2 illustrates custom-made orthotics worn before and during intervention.

The results of changes in the primary and secondary outcome measures are presented in Table 2. Overall, there were significant improvements in all outcomes measured at 1-month and these improvements continued or were maintained at the 3 months timepoint. There was a statistically and clinically significant reduction in pain at 1 month compared to baseline, which continued to improve at the 3-month assessment time point. All other measures, with the exception of self-reported domains of social and school functioning, had significant improvements at 1-month compared to baseline, which were maintained at 3 months assessment, with parental report of child emotional functioning also continuing to improve. At 3-months, the self-reported domains of social and school functioning were significantly improved compared to baseline.

The PGIC scale was used to determine if the participants experienced any change in physiological and psychological well-being related to their lower limb pain including activity limitations, symptoms and overall quality of life since the commencement of the orthotic therapy. The proportion of participants that reported overall health improvement using the PGIC scale at 1-month and 3-months is summarized in Table 3. At 1-month 96% of participants (95%CI: 87%, 100%) reported some improvement and by 3-months all reported improvement (95%CI: 93%, 100%). No participants reported a decline in their overall health and well-being. 

## 4. Discussion

### 4.1. Summary of Findings

This is the first study to report safety and positive outcomes of custom-made orthotics in the management of children with GJH and lower limb pain over a 3 month time period. We found orthotics were well-tolerated and utilized well across the week, and we observed a significant reduction in reported pain, improved HRQoL function and fatigue levels. Significant improvements occurred within one month of wear, which continued to be maintained, or in terms of pain reduction continued to improve at the 3 month timepoint. 

### 4.2. Clinical Implications

There is emerging evidence for the use of prefabricated or off-the shelf orthotics in children with symptomatic GJH [25,26] and custom orthotics in problematic flexible flat foot [44]. Hypermobile patients present with significant foot and ankle deformities that can be very difficult to address [23,45]. Podiatrists have the required skills to modify foot orthotics to improve biomechanical pathologies. Thanks to advances in technology, it is now possible to obtain high resolution scans that can aid in designing custom-made devices for this population group using light, durable and highly resistant materials that improve joint alignments. During this current study, although minor adverse reactions were reported in six participants at the first fitting visit, all had resolved with minimal intervention by the end of this study.

In this study, custom-made orthotics using a 3D mould were fitted to control excessive pronation and capture the exact contour of each participants’ foot shape. Appropriately designed and accurately fitted custom-made orthotics can enhance joint shock absorption, reduce area of excess plantar pressure thus correcting biomechanical alignment and reduce pain while improving functional abilities [46,47]. We observed that in this group of patients with the provision of custom made in shoe orthotics, a reduction in self-reported pain and fatigue and improvement in HRQoL occurred alongside an improved 6MWT distance. Clinicians treating children with symptomatic GJH should consider a podiatric referral for custom orthotics with the goals of reducing pain and fatigue as well as improving walking endurance. 

### 4.3. Strengths and Limitations

The strengths of this study include the detailed methodology on manufacture of the orthotics, global outcomes measured, follow up at two timepoints and sufficient power to show reliable and valid results. There are several limitations inherent to the methodological design of this study. This study was an open before and after design, so therefore limitations include no blinding, randomisation or inclusion of controls. The reason for the use of this study design was to collect evidence on the effects (benefit or harm) of custom-made orthotics in children with GJH and lower limb pain as a foundation for informing the design of future randomised trials by providing an explicit evaluation of the weaknesses of this pre-post intervention study. Nevertheless, due to these inherent study design limitations and potential for bias, findings should be interpreted with caution as the effectiveness of the custom-made orthotic intervention on the outcomes measured cannot be confirmed against a no intervention or alternative intervention control. Furthermore, given that the effectiveness of prefabricated devices in children with symptomatic hypermobility is unknown, future research should focus on determining the effect of prefabricated versus custom-made foot orthotics in randomized, controlled and blinded trials. 

The improvements seen at 1 month continued at 3 months, demonstrating a durable effect over that time period, but this study was limited by the lack of longer follow-up data to establish peak effectiveness and the timepoint at which the therapeutic benefit starts to decrease as this may align with when a child’s feet grow out of that pair of orthotics. Further longitudinal research with a control group study design to determine optimal duration of wear would be required to confirm our findings.

Further studies designed to better understand the mechanisms by which such devices improve outcomes and may influence gait kinematics in children with symptomatic GJH are warranted. In particular, measurement of joint proprioception which is impaired in children with symptomatic GJH [16,48], together with the biomechanics and kinetics of the custom-made orthotics, would be of value. 

## 5. Conclusions

In conclusion, this is the first study to investigate the short to medium term impact of custom-made orthotics in children with GJH and lower limb pain. This study has shown a reduction in pain and improvement in quality of life in hypermobile patients after using custom made orthotics over 3 months. However, despite the growing evidence for the use of pre-fabricated devices in paediatric patients, its effectiveness in hypermobile children still remains unknown. In this study, after wearing custom-made orthotics for 1 month, children reported reduced pain and fatigue while their functional capacity and HRQoL improved. This effect was durable at 3 months, with further improvement in pain. The findings in this study support recommending custom-made foot orthotics for the short to medium term management of symptoms in children with GJH and offer preliminary evidence for conservative podiatric interventions as an adjunct to physiotherapy management of this cohort. Future randomised controlled studies are needed to improve our understanding of the types of orthotics that address specific needs of patients with hypermobility and develop appropriate orthotic prescriptions. For now, these devices appear to offer potential benefits and at least in the short term custom-designed orthotics demonstrate markedly reduced pain and improved HRQoL in children with GJH and lower limb pain.

## Figures and Tables

**Table 1:** Participant demographics and characteristics at baseline prior to intervention.

Characteristic	Study Participants N = 53
Age, years	
Mean (SD)	10.6 (3.7)
Gender, *n* (%)	
Female	35 (66%)
Male	18 (34%)
Beighton score/9	
Mean (SD)	7 (1.24)
Height, m	
Mean (SD)	1.43 (0.18)
Weight, kg	
Median (Range)	33 (20, 82)
Body mass index, kg/m^2^	
Median (Range)	17.2 (12.0, 29.8)
Foot Posture Index (FPI/12 *)	
*Left foot*	
Mean (SD)	9 (2.25)
*Right foot*	
Mean (SD)	10 (2.26)

* A higher score out of 12 indicates a more pronated foot, with the average normative FPI value of +4 (range +1 to +7) for healthy children aged 3–15 years [43].

**Table 2:** Change in primary outcomes and secondary outcome measures over the study period.

Outcome	Mean (SD)			1-Month vs. Baseline		3-Months vs. Baseline		3-Months vs. 1-Month	
	Baseline	1 m	3 m	Mean Diff (95%CI)	*p*-Value	Mean Diff (95%CI)	*p*-Value	Mean Diff (95%CI)	*p*-Value
**Primary outcomes**									
**Pain**									
Visual analogue scale (VAS)/100 mm	55 (17)	29 (21)	22 (18)	−27 (−33 to −21)	<0.001	−33 (−39 to 27)	<0.001	−6 (−11.9 to −0.4)	0.036
**HRQoL**									
Self-reported PedsQL Generic Core Scale Total/100	57 (17)	68 (14)	71 (12)	11 (7.0 to 15)	<0.001	13 (9.4 to 17)	<0.001	2 (−1.6 to 6.3)	0.242
Physical Functioning Domain Score/100	49 (18)	69 (17)	67 (16)	20 (15 to 25)	<0.001	18 (13 to 23)	<0.001	2 (−6.9 to 2.5)	0.352
Emotional Functioning Domain Score/100	60 (23)	68 (21)	71 (19)	8 (3.0 to 14)	0.003	12 (6.2 to 17)	<0.001	3 (−2.2 to 8.7)	0.247
Social Functioning Domain Score/100	68 (19)	73 (20)	75 (17)	4 (−0.9 to 9.7)	0.10	7 (2.0 to 13)	0.007	3 (−2.4 to 8.2)	0.282
School Functioning Domain Score/100	59 (24)	62 (19)	67 (19)	3 (−2.2 to 8.2)	0.25	8 (2.5 to 13)	0.004	5 (−0.5 to 9.9)	0.078
Parent reported PedsQL, Generic Core Scale Total/100	52 (14)	66 (14)	68 (15)	14 (10 to 17)	<0.001	16 (12 to 20)	<0.001	2.3 (−1.4 to 5.9)	0.214
Physical Functioning Domain Score/100	46 (17)	63 (17)	67 (17)	17 (12 to 23)	<0.001	22 (16 to 27)	<0.001	4 (−1.2 to 9.3)	0.127
Emotional Functioning Domain Score/100	50 (19)	62 (19)	68 (19)	12 (6.4 to 18)	<0.001	18 (13 to 24)	<0.001	6 (0.6 to 11.9)	0.030
Social Functioning Domain Score/100	59 (21)	70 (19)	75 (20)	11 (5.8 to 16)	<0.001	16 (11 to 21)	<0.001	5 (0.4 10.5)	0.034
School Functioning Domain Score/100	59 (20)	67 (20)	68 (19)	8.7 (3.7 to 14)	<0.001	9.2 (4.2 to 14)	<0.001	0.5 (−4.4 to 5.4)	0.842
**Secondary outcomes**									
**Functional Endurance**									
Six-Minute walk test (6MWT) meters	477 (68)	503 (57)	510 (59)	27 (18 to 36)	<0.001	33 (24 to 42)	<0.001	6.8 (−2.2 to 15.8)	0.140
**Fatigue**									
Self-reported PedsQL MFS Total Score/100	52 (19)	65 (16)	67 (15)	13 (8.7 to 17)	<0.001	15 (10 to 19)	<0.001	1.8 (−2.2 to 5.8)	0.370
General Domain Score/100	51 (21)	64 (17)	66 (17)	13 (8.2 to 19)	<0.001	15 (9.6 to 20)	<0.001	1.5 (−3.7 to 6.6)	0.581
Sleep/Rest Domain Score/100	51 (21)	64 (18)	67 (20)	13 (7.5 to 18)	<0.001	16 (11 to 22)	<0.001	3.7 (−1.7 to 8.9)	0.177
Cognitive Domain Score/100	56 (23)	64 (18)	67 (18)	8 (3.3 to 13)	0.001	12 (6.7 to 17)	<0.001	3.4 (−1.6 to 8.4)	0.179
Parent reported Peds QL MFS Total Score/100	53 (16)	66 (16)	68 (16)	14 (9.7 to 18)	<0.001	16 (12 to 20)	<0.001	2.2 (−1.8 to 6.2)	0.279
General Domain Score/100	48 (18)	63 (17)	65 (17)	15 (11 to 20)	<0.001	17 (12 to 22)	<0.001	1.5 (−3.2 to 6.2)	0.523
Sleep/Rest Domain Score/100	57 (19)	70 (19)	73 (19)	12 (7.8 to 17)	<0.001	16 (12 to 21)	<0.001	3.9 (−0.7 to 8.4)	0.095
Cognitive Domain Score/100	52 (22)	64 (22)	65 (25)	12 (7.0 to 18)	<0.001	14 (8.2 to 19)	<0.001	1.2 (−4.1 to 6.5)	0.568

Abbreviations. HRQoL: health-related quality of life; PedsQL: Pediatric Quality of life Inventory; PedsQL MFS: Multidimensional Fatigue Scale.

**Table 3:** Patient global impression of change (PGIC).

PGIC *	Participant *n*/N (*n*%)	
	1-Month	3-Months
No change	2/53 (3.8%)	-
Slight Improvement (SI)	8/53 (15%)	10/53 (18.8%)
Much Improved (MI)	29/53 (54.7%)	22/53 (41.5%)
Very Much Improved (VMI)	13/53 (24.5%)	20/53 (37.7%)
Unknown	1/53 (2%)	1/53 (2%)

* No participant reported “slightly worse”, “much worse” or “very much worse”.

## Data Availability

Additional data can be obtained from the corresponding author upon reasonable request.
